# Successful Two-Stage Treatment for Coarctation of the Aorta-postductal Type and Aortic Regurgitation with Thoracic Endovascular Aortic Repair and Aortic Valve Replacement

**DOI:** 10.3400/avd.cr.20-00040

**Published:** 2020-12-25

**Authors:** Ryuta Seguchi, Takafumi Horikawa, Ryuta Kiuchi, Junichiro Sanada, Hiroshi Ohtake, Go Watanabe

**Affiliations:** 1 Department of Cardiovascular Surgery, New Heart Watanabe International Institute, Tokyo, Japan; 2 Department of Vascular Surgery, Ageo Central General Hospital, Saitama, Japan

**Keywords:** coarctation of aorta, thoracic endovascular aortic repair, covered stent

## Abstract

We herein report a case of a 20-year-old man with aortic regurgitation (AR), coarctation of the aorta (CoA), and patent ductus arteriosus (PDA). The preoperative ankle–brachial pressure index was 0.56 in bilateral extremities. Enhanced computed tomography revealed CoA-postductal type. We decided to perform a two-stage surgery: thoracic endovascular aortic repair (TEVAR) for CoA and PDA and then open surgery for AR. TEVAR was successfully performed with deployment of the stent graft at a 31-mm diameter subsequent to balloon dilation. At 8 days after TEVAR, the patient underwent aortic valve replacement via median sternotomy and was discharged without a complication.

## Introduction

Coarctation of the aorta (CoA) is a common congenital heart disease (comprising approximately 6% of congenital heart disease cases). It is commonly associated with persistent ductus arteriosus. Bicuspid aortic valve is observed in nearly two-thirds of infants with CoA. Without treatment, most patients die of stroke, coronary artery disease, or sudden death by the fourth decade of life.^1–4)^

Open surgical repair remains the gold standard therapy for CoA, but the significant collateralization through the intercostal arteries increases the risk of bleeding complications. In addition, other early postoperative complications such as paradoxical hypertension, aortic dissection, left recurrent laryngeal nerve paralysis, phrenic nerve injury, atelectasis, obstructive pneumonia, and subclavian steal syndrome cannot be ignored. In these days, endovascular aortic repair is rapidly becoming the preferred intervention for CoA.^5,6)^ A recent study demonstrated that stenting and surgical repair have the similar efficacy for the treatment of CoA.^7,8)^

We herein present a case of CoA-postductal type associated with aortic regurgitation (AR) due to the bicuspid valve that was successfully treated by a two-stage surgery: thoracic endovascular aortic repair (TEVAR) and aortic valve replacement (AVR).

## Case Report

**Figure 1:**
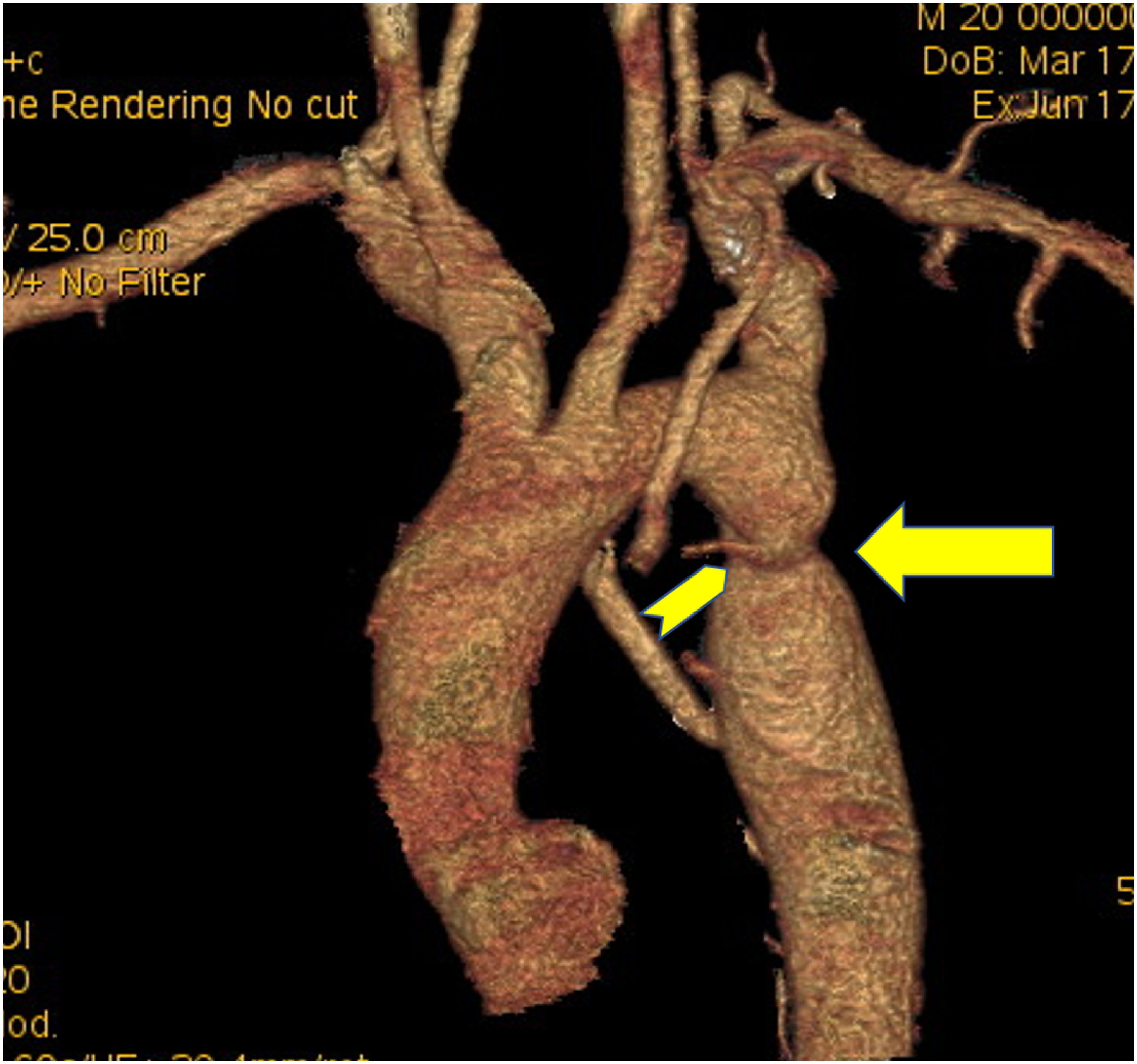
Fig. 1 Three-dimensional computed tomography of the thoracic aorta. The large arrow indicates coarctation of the aorta. The small arrow indicates patent ductus arteriosus located in the proximal portion of the coarctation.

A 20-year-old man was admitted to our hospital with a heart murmur. He had no medical history of traumatic injury or arteritis. On physical examination, his height was 168 cm, and the weight was 65 kg. His pulse rate was 64 bpm, with a blood pressure of 187/69 mmHg in both arms, and all pulses were palpable. However, systolic pressure in the brachial and ankle areas was 80 mmHg, and the ankle–brachial pressure index (ABI) was 0.5. Routine blood tests were normal. A grade 3/6 systolic murmur and a grade 3/4 diastolic murmur were audible along the third left sternum border. Examinations of the respiratory and other systems were normal. Thoracic echocardiography examination indicated grade 4 regurgitation in the aortic valve, due to the bicuspid valve. Severe left ventricular dilatation was observed, with diameters of 74.7 mm and 58.1 mm in diastolic and systolic pressures, respectively. The ejection fraction decreased to 43.6%. The serum brain natriuretic peptide level was 116 pg/mL. In chest radiography, the cardiothoracic ratio was 58%, and rib notching was observed in the inferior surface of the fifth rib. Enhanced computed tomography (CT) indicated a 4.8-mm-diameter strict CoA at the proximal part of the descending aorta (**Fig. 1**). A small-sized patent ductus arteriosus (PDA) was detected in the proximal portion of CoA, with a Qp/Qs ratio of 1.2. Internal mammary and intercostal arteries were dilated as collaterals for lower half of the body.

The diameter of the aorta was 25 mm in the proximal portion of the coarctation and 28 mm in the distal portion of the coarctation.

Treatment for AR, CoA, and PDA was necessary. The one-stage radical operation through anterolateral thoracotomy or median sternotomy can be considered to cause high risk of complications, due to the occurrence of collateral arteries and a large range of aorta. Therefore, a two-staged surgery was performed. We planned to perform TEVAR for CoA and PDA in advance, followed by AVR through median thoracotomy.

**Figure 2:**
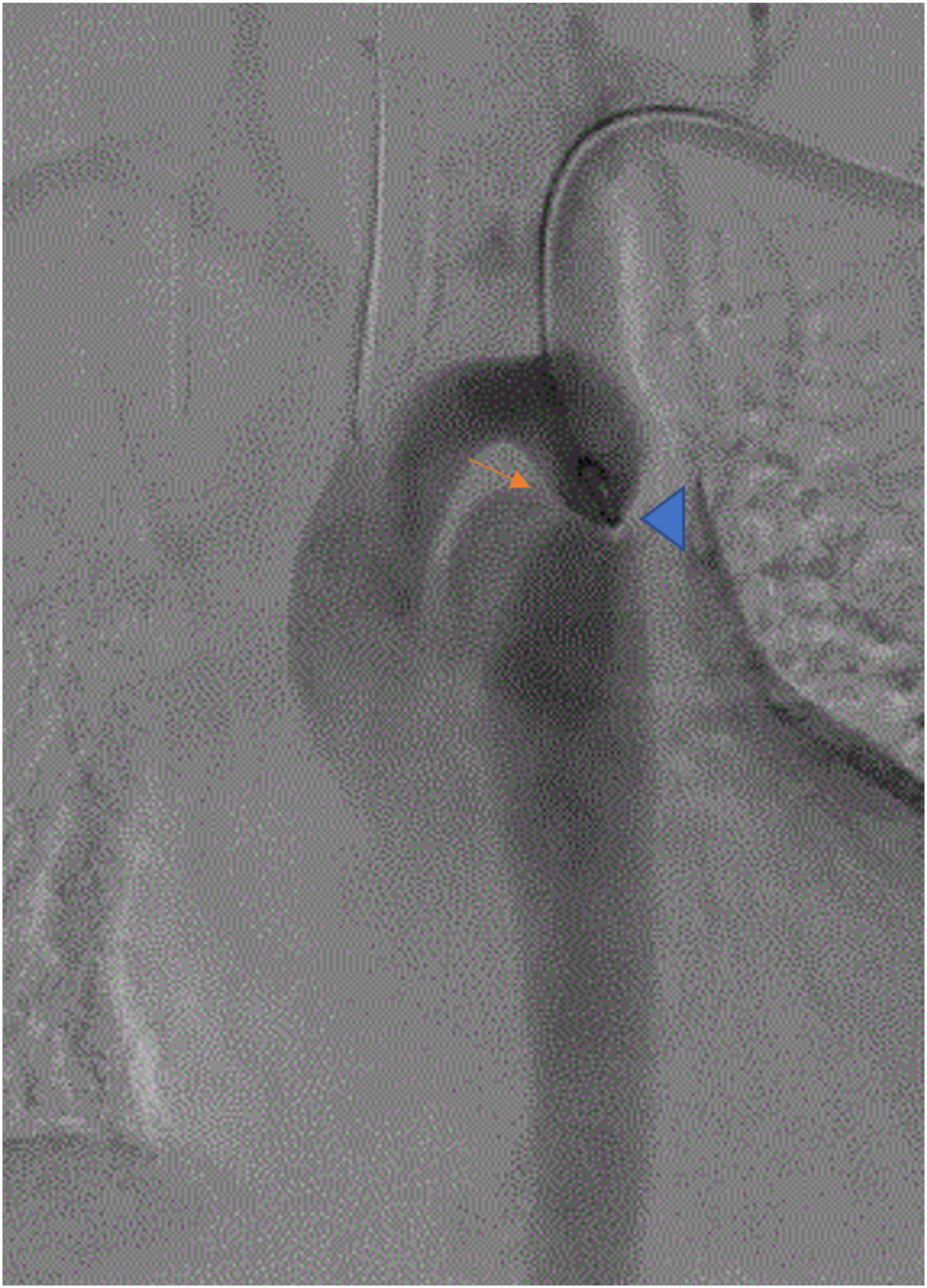
Fig. 2 Primary angiography. Coarcted segment (▶) and patent-ductus-arteriosus flow (➡) are shown.

**Figure 3:**
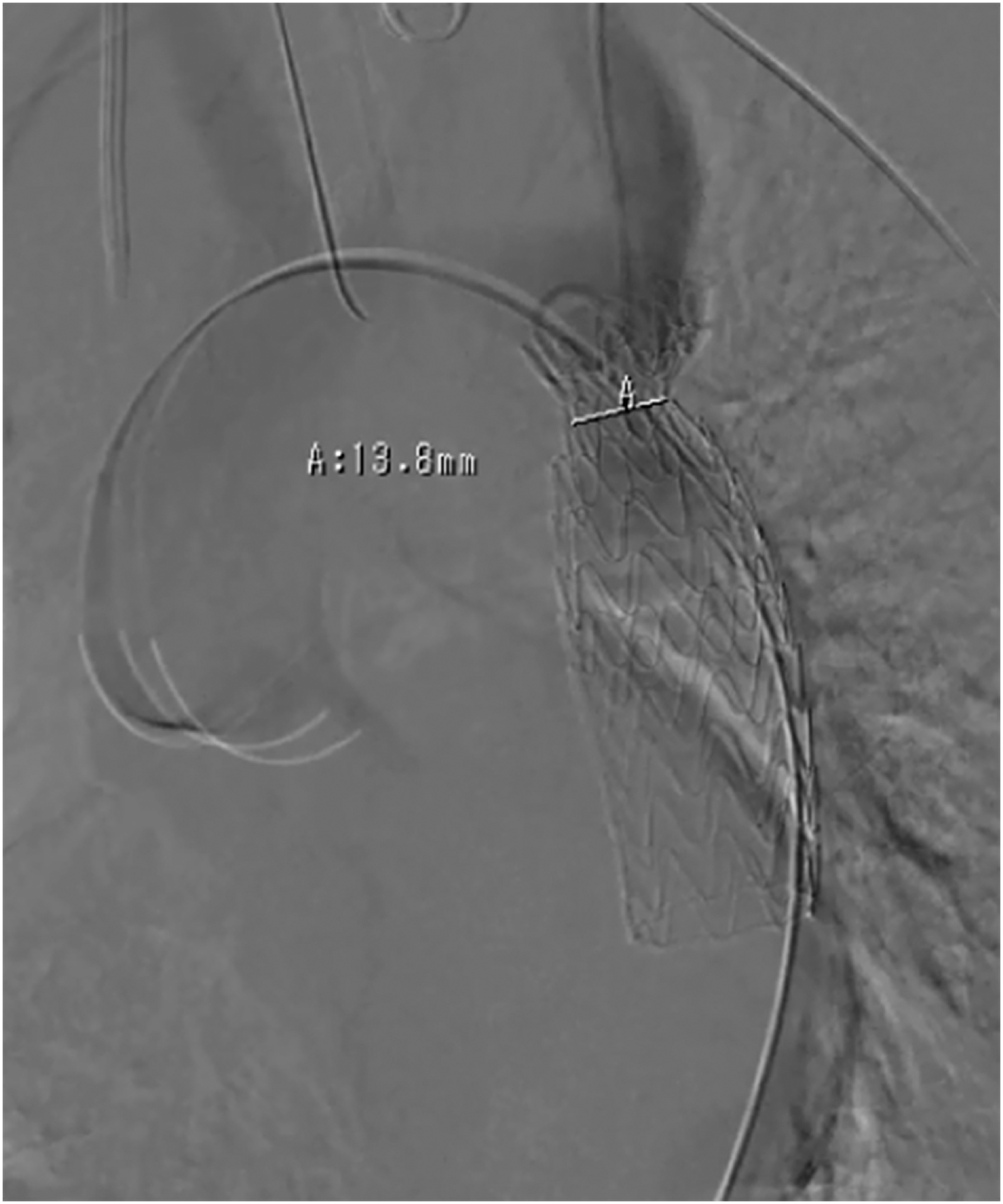
Fig. 3 Final angiography. Coarcted segment (A) was dilated successfully, and disappearance of patent-ductus-arteriosus flow was confirmed.

The right femoral artery diameter was 8.0 mm and was suitable as the access route for TEVAR. The procedure was performed under general anesthesia. The left brachial artery was exposed, and a 4-F pigtail catheter (100 cm, SZ0539) proceeded to the distal arch of the aorta for angiography. Aortography showed a coarcted segment of the descending aorta and PDA flow (**Fig. 2**). A 4-F non-taper angle catheter (Glidecath, Terumo, Tokyo, Japan) and 0.035-in. hydrophilic guidewire (Radiofocus, Terumo Corporation, Tokyo, Japan) were inserted via the right common femoral artery and crossed the coarcted segment. The gradient across the coarcted segment was measured as 80 mmHg. Hydrophilic guidewire was replaced with extra stiff Lunderquist guidewire (Cook, Bloomington, IN, USA). The sheath in the right femoral artery was replaced with 22-F Dryseal sheath (Japan Gore, Tokyo, Japan). The coarcted segment was dilated by 10×40 cm-sized noncompliant balloon catheter (Mustang, Boston Scientific Japan, Tokyo, Japan). However, the pressure gradient remained. Then, a 31×100 mm-sized thoracic stent graft (C-TAG, Japan Gore, Tokyo, Japan) was placed over the coarcted segment and PDA. After confirming the optimal position, the stent graft was released and dilated with a 15×40 mm noncompliant balloon catheter (Maxi-LD, Cardinal Health Japan, Tokyo, Japan). The measured gradient across the coarcted segment after the stent placement was 15 mmHg. Aortography revealed good apposition of the thoracic stent graft and complete exclusion of PDA (**Fig. 3**). No transfusion was required. The patient recovered well, and ABI was 0.90/1.05 postoperatively.

At 8 days after the TEVAR, the patient underwent AVR with the mechanical valve (Carbomedics 27 mm, LivaNova Japan, Tokyo, Japan). Since the afterload had been reduced by the TEVAR, extracorporeal circulation was safely managed, and the heart beat recovery was smooth and uneventful. No transfusion was required. The patient recovered well and was discharged 12 days after AVR. The patient remains asymptomatic. CT performed 3 years postoperatively revealed no aneurysm formation or aortic restenosis.

## Discussion

In this case, less-invasive, safe, and successful treatment was performed for CoA-postductal type and AR by two-stage surgery of TEVAR and AVR. By selecting TEVAR for the treatment of CoA, no hemorrhagic, neurological, or pneumonic complication occurred. Moreover, by reducing the afterload with TEVAR prior to AVR, no ischemic disorder occurred during cardiopulmonary bypass, and the patient was safely weaned from the extracorporeal circulation.

CoA is commonly associated with a cardiac anomaly in the left side of the heart, such as the bicuspid valve, a ventricular septal defect, or mitral valve regurgitation. When the patient comes to the outpatient clinic with such disease and has unexplainable hypertension, CoA should always be considered as the differential diagnosis. If CoA is misdiagnosed, then the patient will die in the fourth decade.

Several options are reported to be the treatment for CoA.^7)^ Open surgery is the golden standard of therapy. Its prognosis is well defined and could be applied at any age. However, risks of complications such as paradoxical hypertension, aortic dissection, left recurrent laryngeal nerve paralysis, phrenic nerve injury, atelectasis, obstructive pneumonia, and subclavian steal syndrome cannot be ignored. Balloon angioplasty is an important less invasive therapeutic option for CoA, which could be applied at any age. However, rates of recoarctation range from 8% to 32%. Furthermore, 24% of patients with balloon angioplasty develop aneurysm. Endovascular stent placement, including TEVAR, is a novel less invasive procedure for CoA. With the use of the covered stent, an extremely less rate of recoarctation and low incidence of pseudoaneurysm are reported.^1,6)^ The fabric within the stent provides additional structural support, creates a protective barrier at the site of stent placement, and reduces the risk of vascular trauma.^6)^ However, because of the required size of the access route and compliance to the growth of body, their use in small children is limited.

The size selection of the device may be controversial.^6)^ In our case, the size of the covered stent was selected according to the diameter of the proximal and distal landing zone, similarly to the treatment for aneurysm. To expand the coarctation, a smaller size than the present case would be sufficient and decrease the risk of aortic rupture. If the device was a stent without cover, a smaller size would be selected. Since the device was a covered stent, hemostasis could be achieved in case of rupture. Therefore, we chose a 31-mm-diameter stent graft to fit the distal landing zone that was 28 mm in diameter. We also intended to cover PDA. Therefore, we decided that the adequately large covered stent is the best choice.

The proximal landing zone would be indicated as short in this case. Since the main purpose of the procedure was to expand the coarctation and additional stent graft should still be inserted in the proximal area, the length of proximal landing zone was only 12 mm. The bird beak sign in the minor curvature can also be avoided to prevent radial force to the major curvature and covering the left subclavian artery. In case of rupture, an additional stent graft to the proximal portion and Zone 2 TEVAR were performed. If PDA could not be closed by the stent graft, we planned to close it during AVR by opening the main trunk of the pulmonary artery; in fact, PDA was successfully closed by the stent graft. Additional surgical options should be crucially prepared while this procedure is performed with confidence in case of emergency.^5)^

With regard to balloon expansion, overexpansion should be avoided to prevent aortic divulsion. For a 4.8-mm-diameter coarctation site, 10 and 15 mm balloons were used for the initial expansion and poststenting expansion. The sizes were smaller than those reported by Mustafa et al.^3)^ Further expansion may had been acceptable in this case; however, due to the risk of rupture, a smaller-sized balloon was used, and the procedure ended safely. The pressure gradient of 15 mmHg remained postintervention; however, satisfying clinical outcomes were achieved.

In the present case, the access route for TEVAR was safely ensured from the femoral artery. However, in some cases of CoA, arteries in the lower extremities are exposed to low blood pressure for years, so that the femoral artery diameters are small. Korkmaz et al. reported a successful endovascular treatment for CoA and PDA by the retroperitoneal iliac approach.^2)^ An alternative access route for the femoral artery is sometimes required.

## Conclusion

Although its long-term prognosis is unknown, TEVAR has the potential to be the most preferable treatment for adult CoA. In cases with cardiac disorder, a two-stage operation should be considered.

## Additional Note

Informed consent was obtained from the patient for submitting this paper.

## References

[1] Torok RD, Campbell MJ, Fleming GA, et al. Coarctation of the aorta: management from infancy to adulthood. World J Cardiol 2015; 7: 765-75. 26635924 10.4330/wjc.v7.i11.765 PMC4660471

[2] Korkmaz O, Beton O, Goksel S, et al. Thoracic stent graft implantation for aortic coarctation with patent ductus arteriosus via retroperitonial iliac approach in the presense of small sized femoral artery. Case Rep Cardiol 2016; 2016: 7941051. 27242935 10.1155/2016/7941051 PMC4868890

[3] Batur MK, Koç M, Ozer I, et al. Usefulness of endovascular stent-graft for combination with a strict aort coarctation and patent ductus arteriosus for an adult patient: a case report. Cases J 2009; 2: 8477. 20181210 10.4076/1757-1626-2-8477 PMC2827084

[4] Schneider H, Uebing A, Shore D. Modern management of adult coarctation: transcatheter and surgical options. J Cardiovasc Surg (Torino) 2016; 57: 557-68. 27243624

[5] Erben Y, Oderich G, Verhagen HJM, et al. Multicenter experience with endovascular treatment of aortic coarctation in adults. J Vasc Surg 2019; 69: 671-9.e1. 30528403 10.1016/j.jvs.2018.06.209

[6] Lala S, Scali ST, Feezor RJ, et al. Outcomes of thoracic endovascular aortic repair in adult coarctation patients. J Vasc Surg 2018; 67: 369-81.e2. 28947226 10.1016/j.jvs.2017.06.103

[7] Roselli EE, Qureshi A, Idrees J, et al. Open, hybrid, and endovascular treatment for aortic coarctation and postrepair aneurysm in adolescents and adults. Ann Thorac Surg 2012; 94: 751-8; discussion, 757-8. 22704801 10.1016/j.athoracsur.2012.04.033

[8] Carr JA. The results of catheter-based therapy compared with surgical repair of adult aortic coarctation. J Am Coll Cardiol 2006; 47: 1101-7. 16545637 10.1016/j.jacc.2005.10.063

